# Utility of Milk Coagulant Enzyme of *Moringa oleifera* Seed in Cheese Production from Soy and Skim Milks

**DOI:** 10.3390/foods6080062

**Published:** 2017-08-05

**Authors:** María Alejandra Sánchez-Muñoz, Mónica Andrea Valdez-Solana, Claudia Avitia-Domínguez, Patricia Ramírez-Baca, María Guadalupe Candelas-Cadillo, Miguel Aguilera-Ortíz, Jorge Armando Meza-Velázquez, Alfredo Téllez-Valencia, Erick Sierra-Campos

**Affiliations:** 1 Facultad de Ciencias Químicas, Universidad Juárez del Estado de Durango, Av. Artículo 123 S/N Fracc. Filadelfia, Gómez Palacio, Durango, CP 35010, Mexico; airama33@gmail.com (M.A.S.-M.); valdezandyval@gmail.com (M.A.V.-S.); ramirezbp2000@hotmail.com (P.R.-B.); candelascadillo@gmail.com (M.G.C.-C.); maguilerao@hotmail.com (M.A.-O.); jorgemezav68@gmail.com (J.A.M.-V.); 2 Facultad de Medicina y Nutrición, Universidad Juárez del Estado de Durango, Av. Universidad y Fanny Anitua S/N Col. Centro, Durango, Dgo, CP 34000, Mexico; avitiaclaudia@gmail.com (C.A.-D.); tellezalfredo@gmail.com (A.T.-V.)

**Keywords:** soy milk, milk-coagulant activity, *Moringa oleifera*, seeds

## Abstract

In this study, the potential use of *Moringa oleifera* as a clotting agent of different types of milk (whole, skim, and soy milk) was investigated. *M. oleifera* seed extract showed high milk-clotting activity followed by flower extract. Specific clotting activity of seed extract was 200 times higher than that of flower extract. Seed extract is composed by four main protein bands (43.6, 32.2, 19.4, and 16.3 kDa). Caseinolytic activity assessed by sodium dodecyl sulphate-polyacrylamide gel electrophoresis (SDS-PAGE) and tyrosine quantification, showed a high extent of casein degradation using *M. oleifera* seed extract. Milk soy cheese was soft and creamy, while skim milk cheese was hard and crumbly. According to these results, it is concluded that seed extract of *M. oleifera* generates suitable milk clotting activity for cheesemaking. To our knowledge, this study is the first to report comparative data of *M. oleifera* milk clotting activity between different types of soy milk.

## 1. Introduction

Nowadays, foods are intended not only to satisfy hunger and provide necessary nutrients for humans, but to prevent nutrition-related diseases and improve consumers’ physical and mental well-being [1]. Moreover, there is plenty of scientific literature that demonstrates the close connection between diet and health, particularly related to chronic diseases, which have encouraged the development of growing spectrum products such as nutraceuticals, medifoods, and vitafoods [2,3].

In México, data from three national surveys conducted in 1988, 1999, and 2006, using the International Obesity Task Force classification system, described the upward trends on overweight and obesity in school-age children and teenagers at a national level [4]. Besides, increased world population, and the continuous rise of morbid obesity and other nutritional diseases have led to the employment of protein from vegetal sources, along with the preference of low-fat dairy products in consumers from our country. Therefore, people are constantly pursuing better life quality by eating low-fat dairy products which may reduce the risk of stroke or coronary heart disease [5]. Also, the remarkable sales in soy-based products can be attributed to the beneficial health properties of soy-derived foods.

Cheese is a widely consumed product by the general population, it is highly-concentrated, rich in proteins and lipids, essential amino acids, and minerals such as calcium and phosphorus. The first step in cheesemaking is the milk clotting process where κ-caseinolytic enzymes contribute to micelle aggregation, usually performed by animal rennet, which has been the traditional coagulant in cheese manufacture for centuries [6]. However, dairy products have recently come under fire from researchers showing the detrimental effects of high saturated fat and cholesterol concentrations to the body [7]. Since full-fat dairy products contain more calories, many experts assumed that avoiding them would lower the risk of diabetes. However, some studies have found that yogurt intake, but not milk, is consistently associated with lower incidence of diabetes mellitus. On the other hand, cheese intake, despite the higher calories, fat, and saturated fat content, is also associated with lower diabetes risk in several but not all studies [7,8,9]. These findings suggest that health effects of dairy products may depend on multiple complex characteristics and represent promising areas for further research [10]. Although the data are controversial, Mexican gastronomy is innovating products (lactose-free, high calcium, and weight-control foods) using vegetables harvested in large amounts in Mexican soil.

Nutritionists have mentioned that the incorporation of bioactive components in dairy products might confer several advantages [11]. For example, phenolic compounds have been extracted from a variety of plant sources and used as matrix food ingredients improving functional properties of dairy products such as storage and heat stability, as well as foaming properties [12,13]. Dairy product enrichment with phenolic compounds has been proposed for beverages [13], yogurt [14], milk powder [15], and processed cheese [16]. Given that polyphenols interact with proteins [17], their addition to milk may result in a high yield recovery in cheese, mainly attributed to hydrophobic and hydrophilic interactions [18] that depend on pH, molar ratio, and molecular properties of the polyphenols [15]. However, recent studies have shown that rennet-induced coagulation is altered by the addition of tea polyphenols to milk [19,20] but in spite of this limitation, cheddar type cheese has been produced from milk enriched with green tea extract [21]. In addition, plant extracts could increase the shelf life of dairy products by inhibiting oxidation of polyunsaturated fatty acids and the development of off-flavor aromas [22].

Yadav et al. [23] stated that soy milk is a well-known protein enriched bio-functional food, but its acceptability is reduced by the presence of complex sugars which give a bean-like flavor. However, it was shown that fermentation produces a reduction of such off-flavor compounds. Hence, soy by-products such as yogurt and cheese can be nutraceutical products with antioxidant potential.

It has been reported that commercial plant proteases, such as bromelain and papain, can clot the protein in soy milk forming a curd [24]. Unfortunately, unlike casein in bovine milk, enzymatic curdling of soy milk produces poor flavor and texture since proteolysis is more pronounced in cheese processed with vegetable coagulants which leads to a soft and buttery cheese texture and, partly, to liquefaction and loss of shape [25,26]. In addition, short peptides produced by its high proteolytic activity affect the flavor, which results in an excessively acidic and bitter cheese [27]. For this reason, the commercial use of bromelain, ficin, and papain to clot soy milk has not been successful [28,29,30]. However, a more recent study showed that *Saccharomyces bayanus* SCY003 protease produced a soy curd with elasticity that resembles milk-casein cheese [31]. Therefore, it is important to continue the search of proteases with the capability to coagulate soy milk and improve the acceptability of soy cheese.

*Moringa oleifera* is grown in rural regions of Mexico and its different parts, such as leaves, flowers, and seeds, are edible. It is a source of protein, calcium, iron, carotenoids, and phytochemicals, and it is employed for several applications in developing countries [32]. Previously, it has been reported that *M. oleifera* is an interesting source of milk clotting enzyme. Pontual et al. [33] reported the caseinolytic and milk-clotting activities of *M. oleifera* flowers using azocasein and skim milk as substrates, respectively. *M. oleifera* seed extract was also used as a milk-clotting agent, and the resulting curd was white and firm [34]. Despite the aforementioned studies on milk-clotting enzyme from *M. oleifera*, a deep evaluation of this potential source of a rennet substitute is still absent. In addition, there are no studies that evaluate the efficiency of *M. oleifera* proteases to clot soy milk proteins. Thus, the aim of this research was to determine the potential ability of different parts from *M. oleifera* to coagulate whole, skim, and soy milks and to investigate the use of *M. oleifera* in the production of soft cheese.

## 2. Materials and Methods

### 2.1. Vegetal and Dairy Material

*Moringa oleifera* seeds, flowers, and leaves were obtained from Lombardia, Michoacán, located at 19°01′30″ N and 102°05′39″ W. All the samples were dried and crushed at room temperature and stored in closed containers at −20 °C. Commercial cow whole and skimmed milk (Lala^®^ and Carnation^®^, Nestle^®^, respectively) and soy milk powders (Soyapac^®^, Colpac; AdeS^®^, and Soyalac^®^) were used to evaluate different parameters related to the milk coagulant enzyme (MCE).

### 2.2. Enzyme Extraction

Briefly, duplicate samples of 2 g of powder of the different parts of the plant were immersed in 10 mL of 50 mM phosphate buffer, pH 7.0 (1:10; *w*/*v*), the mixtures were macerated by two methods: traditional stirring for 4 h and ultrasonic bath (42 KHtz) for 15 min at room temperature. The extracts were centrifuged at 3500 rpm for 10 min. Finally, the supernatants were stored at 4 °C until used.

### 2.3. Milk-Clotting Activity (MCA)

The milk-clotting activity was determined following the procedure described by Arima et al. [35] with some modifications. Briefly, a suspension of skim milk powder was used as a substrate with 10 mM CaCl_2_, pH 6.5. The milk was previously incubated at 35 °C for 5 min, then MCE was added at a 10:1 ratio (*v*/*v*, skim milk to enzyme extract). One unit of milk-clotting activity (MCA) is defined as the amount of enzyme to clot 1 mL of a solution containing 0.1 g skim milk powder in 40 min at 35 °C.

The time between the addition and the appearance of clots was registered and the total MCA was calculated as follows:

(1) $$MCA\;\left(\frac{SU}{mL}\right)=\frac{2400}{Coagulation\;time\;\left(s\right)}\times dilution\;factor$$

In order to compare the efficiency of the milk clotting enzyme, rennet from calf stomach (mixture of chymosin and pepsin) from Sigma-Aldrich (Toluca, Mexico) and 5% NaCl were used as the positive and negative controls, respectively. The specific activity was determined by dividing the MCA between protein concentrations of extracts [Soxhlet units (SU)/mg protein].

### 2.4. Caseinolytic Activity

Caseinolytic activity was measured by Sarath’s method [36]. Suitably diluted seed extract solution was added to 2.5 mL of 1% casein dissolved in 50 mM NaH_2_PO_4_ (pH 6.5). The mixture was incubated at 37 °C for 30 min and then 2.5 mL of 5% trichloroacetic acid solution (TCA) was added. After precipitation, all mixtures were centrifuged at 10,000 rpm for 20 min and the absorbance of the supernatant at 280 nm was registered. The blank sample was prepared in the same way by adding TCA prior to the addition of the substrate. One unit of caseinolytic activity of enzyme was defined as the amount of enzyme that delivers 1 µg of tyrosine (Tyr) and causes a 0.01 increase in absorbance at 280 nm through 1 cm of cuvette path length. Rennet from calf stomach (mixture of chymosin and pepsin) from Sigma-Aldrich and 5% NaCl were used as positive and negative controls, respectively.

### 2.5. Protein Determination

The protein concentration was determined according to Lowry et al. [37]. A standard curve was generated using bovine serum albumin (10–500 µg/mL) as the standard. Alkaline copper sulphate reagent was added to the different dilutions and sample solutions, which were incubated at room temperature for 10 min. Then, Folin & Ciocalteu’s reagent (commercially available, F9252 from Sigma-Aldrich) was added to each tube and incubated for 30 min. The mixture absorbance was measured using a spectrophotometer (600 nm, Hach DR 5000, USA Hach, Loveland, CO, United States.

### 2.6. Sodium Dodecyl Sulphate-Polyacrylamide Gel Electrophoresis (SDS-PAGE)

The crude extract from *M. oleifera* seed was characterized by sodium dodecyl sulphate-polyacrylamide gel electrophoresis (SDS-PAGE) [38]. The crude extract was first boiled for 5 min in the presence of SDS and β-mercaptoethanol and 50 μg/mL of protein concentration was loaded into the gel (5% of stacking gel and 12% of separating gel). SDS-PAGE was run at 120 volts (Miniproteam II cell, Bio-Rad, Hercules, CA, USA) until the bromophenol blue dye marker disappeared from the separating gel. Proteins were stained with Coomassie blue R250 and washed with methanol/acetic acid/water (40:50:10) solution to remove the dye excess. A low molecular weight marker was used in a range of 20 to 110 KDa as the standard (Bio-Rad).

### 2.7. Cheese Elaboration

Cheesemaking was carried out by preheating portions of 1000 mL of skim and soy milks at 60 °C. Then, 5 mL of CaCl_2_ (2 M, pH 6.5) was added only to skim milk, and 50 mL of seed crude extract (10 g/100 mL of milk) or renin (0.14 mg/mL) were incorporated into the mixture. After, both milks were incubated for 60 min at 50 °C, the curd was cut and stirred at 150 rpm in an orbital shaker, and then the whey were drained. Finally, the curds were placed in round-bottomed containers and cheeses were stored in polyethylene bags at 4 °C.

### 2.8. Statistical Analysis

The data of three independent experiments were collected and statistically analyzed using one-way analysis of variance (one-way ANOVA), followed by Tukey’s honestly significant difference (HSD). Probability *p* < 0.05 indicated statistically significant differences.

## 3. Results and Discussion

Plant enzymes are widely studied as potential coagulants in cheese production, for example, extracts of *Cynara scolymus* L. [39], *Albizia lebbeck* [40], *Centaurea calcitrapa* [41], the latex of *Sideroxylon obtusifolium* [42], the flowers of *Silybum marianum* [43], *Cynara scolymus* [44], and *Jacaratia corumbensis* O. Kuntze [45]. Ginger rhizome has been used as a source of milk coagulating clotting cysteine protease [46]. However, *Cynara cardunculus* L. extract is particularly popular due to its traditional use in elaborating artisanal sheep milk cheese [25]. In contrast, Mexican plants with milk-clotting activity have been scarcely studied. It has been reported that *Solanum elaeagnifolium* berry extract can be suitable for soft cheese manufacturing, for example for cream cheeses [47]. Therefore, generating knowledge and expanding the field of Mexican natural coagulants is of great importance.

### 3.1. Initial Analysis of Coagulant Activity in Different Parts of the M. oleifera

**Figure 1:**
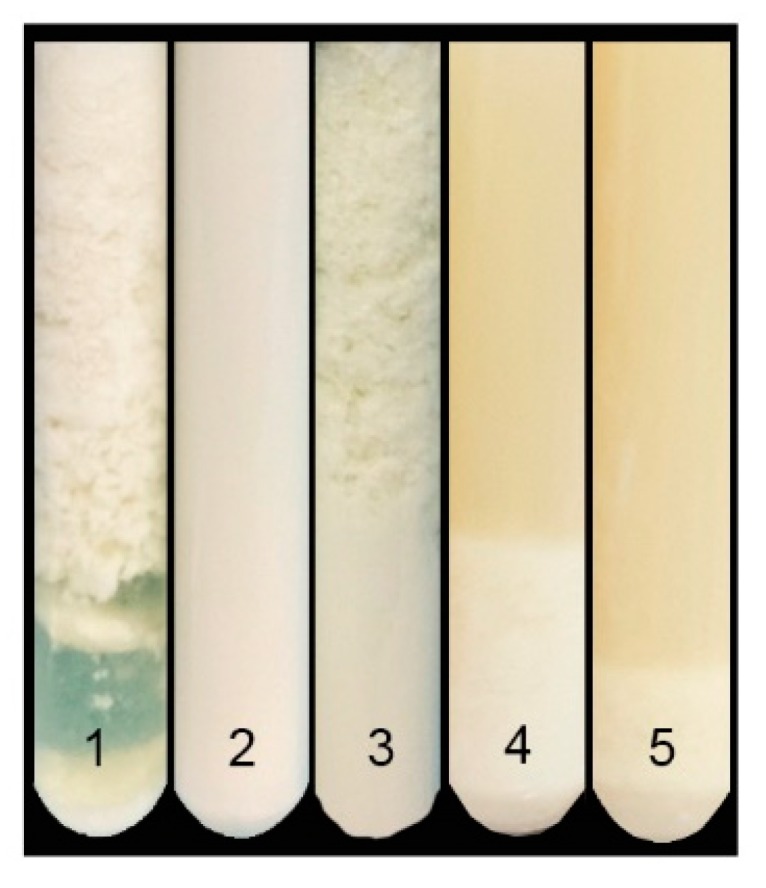
Test tubes of curd formation using *M. oleifera* crude extracts on whole milk. 1: Calf rennet (positive control); 2: NaCl 5% (negative control); 3: Seed extract; 4: Flower extract; 5: Leaf extract.

Previous studies have described milk clotting activity in different *M. oleifera* tissues [33,34]. To gain a more complete picture of the tissue-level localization of milk clotting activity in *M. oleifera*, we prepared extracts from seeds, flowers, and leaves. As summarized in Figure 1, the coagulant activity of each extract was studied using whole cow’s milk as the substrate. Commercial rennet (tube 1) and 5% of NaCl (tube 2) were used as positive and negative controls, respectively. Seed extract showed high clotting activity (tube 3), while milk clotting activity was only slightly detected for flower extract (tube 4) and was absent for leaf extract (tube 5). The total milk clotting activity of the seed extract was 3419 SU/mL, which corresponds to 50% of the total activity obtained with calf rennet, however, in terms of specific activity only represents a 30% decrease (Table 1). These data are consistent with those reported by Talajsir et al. [34] for seeds only, but were not similar for other tissue extracts (leaves and flowers). The activity with flower extract was 13.66 SU/mL, which agrees with the activity reported by Pontual et al. [33]. It is noteworthy that milk clotting activity of seed extract does not seem to depend on the milk type (whole or skim milks) and along with a previous background where similar activity values were reported on skim milk, our *M. oleifera* seed extract has approximately 8 times more activity. Therefore, we chose seed extract for the following experiments.

### 3.2. Electrophoretic Pattern of Moringa oleifera Extracts

**Figure 2:**
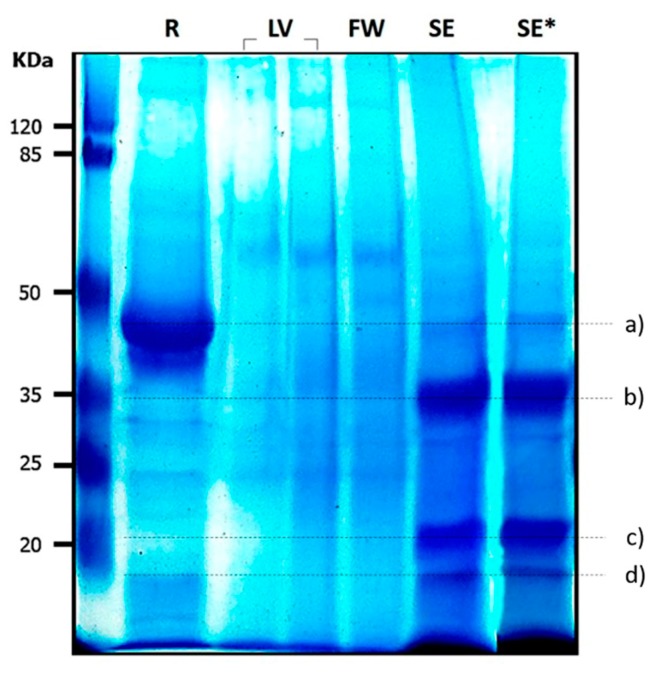
Electrophoretic pattern of *Moringa oleifera* crude extracts. R: Rennet, LV: Leaves, FW: flowers, SE: Seed extract, SE*: Seed extract macerated with ultrasonic bath. (**a**–**d**): Proteins of interest. Data are representative of three experiments.

Electrophoretic patterns of each extract were evaluated to determine the molecular weight of proteins with greater abundance that possibly participate in milk coagulation. The results of analysis by SDS-PAGE of *M. oleifera* extracts are shown in Figure 2. A comparison of protein content of different crude extracts shows the variable protein levels in leaves, flowers, and seeds. Both leaves’ and flowers’ protein patterns showed some discrete bands. On the other hand, predominant bands were found in seed crude extract. From molecular marker and sample protein, an electrophoretical mobility plot was made in order to determine the molecular weight of seed extract proteins. There were four main bands (a to d) and their apparent molecular mass were 43.6, 32.2, 19.4, and 16.3 kDa. Some bands observed in the extract had similar molecular weights as reported for calf rennet (mixture of chymosin and pepsin) and exhibited one prominent band of 48 kDa, suggesting that *M. oleifera* seeds may possess one or more enzymes with rennet-like activity. However, more studies on the structural characterization of these proteins are needed since lectin, an acid protein with 30 kDa, from *M. oleifera* seeds has coagulant properties which are mainly used to reduce water turbidity [48,49].

### 3.3. Effect of Substrate and Enzyme Concentration on Skim Milk Clotting Activity

Measuring enzyme and substrate concentration, in terms of observed activity, is a key task to determine the quality of the milk coagulation process. It is well known that milk sources and enzyme types significantly affect the cheese yield and curd formation time [50]. Therefore, it is necessary to explore the effect of these parameters to control the hydrolysis of casein by *M. oleifera* seed extract.

**Figure 3:**
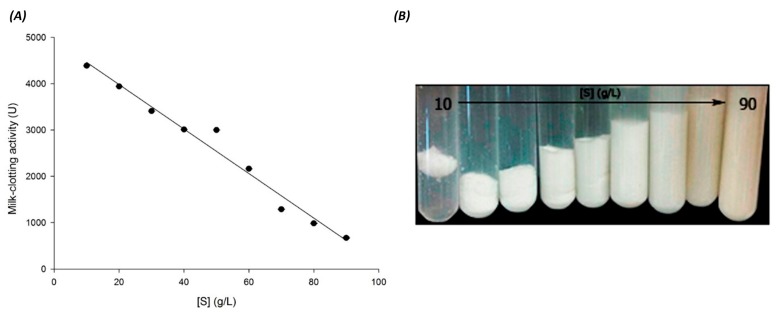
Substrate concentration effect on skimmed milk-clotting activity of seed extract. (**A**): Left plot was drawn using the data obtained from the milk-clotting activity assay. (**B**): Right image shows the curd formation in test tubes. One unit of milk-clotting activity (MCA) is defined as the amount of enzyme to clot 1 mL of a solution containing 0.1 g skim milk powder in 40 min at 35 °C. Substrate concentration is expressed as [S]. All experiments were performed in triplicate, and each data point represents the means of at least three determinations. The regression line equation is (y = −48.03x + 4941.39) with a correlation coefficient of *R* = 0.99.

In order to evaluate the effect of substrate concentration on milk-clotting activity, skim milk varied from 10 to 90 g/L. The use of this range of substrate concentrations allowed an accurate determination of time at the onset of lump formation. However, it is important to mention that it was almost impossible to measure the time required for coagulation when a low concentration of substrate was used (<10 g/L) because the concentration of 20 mg/mL of enzyme (seed extract) caused an instant coagulation; on the other hand, when a higher concentration of substrate was used, there was a marked increase in milk coagulation time. This is probably due to the increase in the viscosity of the reaction mixture. As shown in Figure 3, the milk-clotting activity decreased as substrate concentration was increased in a directly proportional manner until the concentration of substrate reached about 90 g/L. In addition, according to the clotting time present in these experiments, it was observed that 10 g/L of substrate, coagulated at 35 °C and pH 6.5 with CaCl_2_, presented a clotting time of 5.47 ± 0.33 s. The reaction mixture with 90 g/L of substrate had a clotting time of 35.74 ± 10.04 s, which is 6.5-fold higher. Thus, our results revealed that the optimum substrate concentration was 10 g/L, suggesting that a high concentration of skim milk has an adverse effect over the catalytic efficiency. These results do not agree with those reported by Ahmed and Helmy [51] for *Aloe variegate* and *Bacillus licheniformis* 5A5 milk-clotting enzymes, who report that increasing skim milk concentration caused a significant increase in MCA up to 60 g/L for both enzymes. However, Wahba et al. [52] reported that substrate concentration over 60 to 210 g/L increased the clotting time. In addition, a study with *Mucor pusillus* pepsin showed a gradual decrease in milk clotting activity to 36.5% with skim milk concentration reaching the 200 g/L [53]. In any case, such retardation of milk-clotting by seed extract may be attributed to several factors. There is an insufficient amount of substrate (casein molecules) due to dilution which restricts the ability of the enzyme to act at its full capacity [54]. The increased viscosity of the solution at higher concentrations of milk diminishes the enzyme activity or it may be due to the smaller amount of hydrolyzed κ-casein when coagulation is initiated; both facts have been stated by Dalgleish [55] and Low et al. [56].

**Figure 4:**
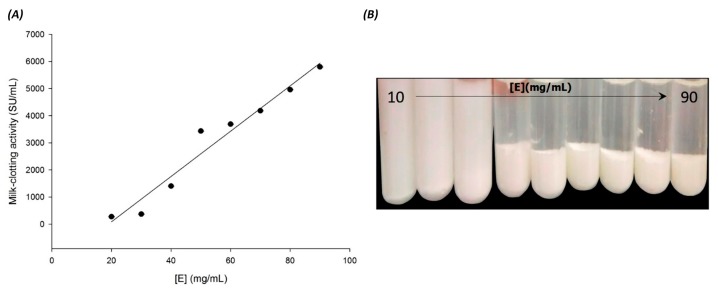
Enzyme concentration effect on skimmed milk-clotting activity of seed extract. (**A**): Left plot was drawn using the data obtained from the milk-clotting activity assay. (**B**): Right image shows the curd formation in test tubes. One unit of milk-clotting activity (MCA) is defined as the amount of enzyme to clot 1 mL of a solution containing 0.1 g skim milk powder in 40 min at 35 °C. Enzyme concentration is expressed as [E]. All experiments were performed in triplicate, and each data point represents the means of at least three determinations. The regression line equation is (y = 83.55x + (−1582.85)) with a correlation coefficient of *R* = 0.98.

The influence of enzyme concentration is an important parameter that affects cheese quality and yield. The effect of enzyme concentration on the milk coagulant activity of seed extract is shown in Figure 4. At a lower concentration of enzyme (<20 mg/mL) in 10 mL of the skim milk, the seed extract did not show any sign of coagulation for two or more hours at 35 °C. The relative activity increased linearly from 20 to 90 g/mL, suggesting that the maximum milk-clotting activity is reached when the enzyme concentration is up to 90 mg/mL. From the results, it can be deduced that a proportional relationship between the enzyme concentration and milk clotting activity exists. However, greatly diluted enzyme did not have enough activity to coagulate the skim milk. These results agree with those reported by other authors, who have mentioned that the clotting time decreased as the concentration of enzyme increased [51,57,58]. Lopez et al. [59] and Najera et al. [58] attributed this phenomena to a higher level of κ-casein proteolysis. On the other hand, at lower concentrations of enzyme, the activity decreased due to the insufficient amount of enzyme to clot the milk. It is noteworthy that milk coagulation by rennet combines an initial enzymatic hydrolysis reaction and a subsequent enzyme-independent protein aggregation reaction [60].

### 3.4. Effect of Enzyme Concentration on Caseinolytic Activity

The dairy industry characterizes rennet enzyme using two parameters. The first is the milk-clotting activity (MCA) expressed in International Milk-clotting units, determined by a standard method [48] that describes the ability to aggregate micelles by cleaving the Phe105-Met106 bond or a nearby bond of κ-casein. The second property is the general proteolytic activity (PA), which is the ability to cleave any bond of casein [61]. The MCA/PA ratio captures the essential quality of a milk-clotting enzyme to cheese’s elaboration.

**Figure 5:**
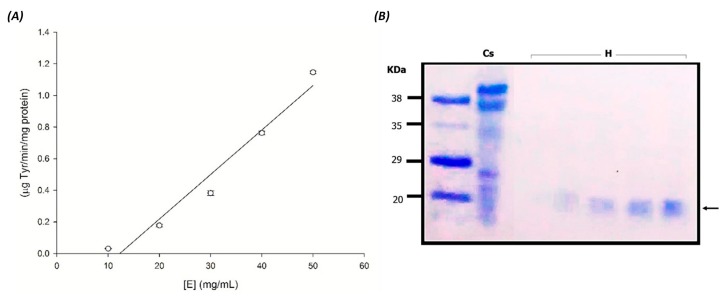
Effect of enzyme (seed extract) concentration on caseinolytic activity. (**A**): Left plot was drawn using the data obtained from the casein hydrolysates with different enzyme concentrations ([E]: 10, 20, 30, 40, and 50 mg/mL). (**B**): Right image shows SDS-PAGE of Cs: 1% Casein; and H: casein hydrolysates. All experiments were performed in triplicate.

Figure 5 shows the effect of seed extract concentration (enzyme) in the assays from 10 to 50 (mg/mL) on the rate of caseinolytic activity. The proteolytic activity was measured by the casein digestion method. It can be observed that the PA is dependent of the enzyme concentration. This result suggests that seed extract has an excellent catalytic property. In addition, the MCA/PA ratio (2995:1) for seed extract was observed, and it was much higher than that reported in most previous studies [33,34]. The high quality of the enzyme in *M. oleifera* seed extract seems to be a promising asset for industrial purposes.

### 3.5. Composition of Soy Milk

Many methods have been developed to measure the activity of milk clotting enzymes. Most are based on the time necessary to coagulate a casein-based substrate. These methods measure the enzymatic and non-enzymatic reactions in milk coagulation. The work of Zhao et al. [62], based on the Arima et al. [35] study, determined MCA, and the results were expressed in Soxhlet units. However, it is not clear if these methods are also regularly used to measure the coagulant activity of plant extracts with other types of milk such as soymilk. Therefore, we decided to assess if the constituents of different soy milks, such as protein and fat content, fiber, carbohydrates, and some minerals, may influence the milk clotting process and, consequently, the finished cheese.

Soy milk is a highly diverse fluid consisting of a vast number of substances, the main ones being soluble carbohydrates (sucrose, raffinose, stachyose, others), protein, fiber, minerals, and fat [63]. Soy milk has approximately 20% lipids but this concentration varies among regions. Soybeans harvested in the United States have more lipids than those from China. Thus, the actual composition of soy milk depends on many factors, including varieties, growing season, geographic location, environmental conditions, and methods of making soy milk (nama-shidori and kanetsu-shibori) [64].

Soy milk is an emulsion composed of soy protein, mainly glycinin and β-conglycinin which represent 60% of total soy milk protein, and lipids, mainly triacylglyceride composed of linoleic acid, oleic acid, and phospholipids. In addition, phytic acid, minerals, and oligosaccharides are present in the soluble component of the emulsion and when some bivalent ions such as calcium and magnesium are added to soy milk in order to elaborate tofu, the ions combine with phytic acid [65]. As a result, the pH decreases and the protein immobilizes, forming a large body constructed with proteins, lipids, and minerals [66]. 

The chemical compositions of the different types of soy milk used in this study are presented in Table 2. From the results, all types of soy milk were mainly composed of protein and fat. However, a lower percentage of nutritional composition was found for Soyalac. To elaborate firm tofu, the average ratio of fat-protein is 0.55 to 1. The ratios for the different soy milks AdeS, Soyalac, and Soyapac were 0.72, 0.5, and 0.9, respectively due the protein content variations in commercial soy milk ranging from 2.0 to 6.6 g, while total fat content also varied from 1.0 to 4.5 g. These data suggest that Soyalac maintains the correct ratio to produce a cheese like firm tofu. However, the process of soy milk manufacturing might cause chemical changes, which leads to soy protein degradation. Therefore, it is known that the physicochemical properties of soy milk play an important role in tofu making. In addition, if the MCA of *M. oleifera* seed extract accepts different substrates, the question arises as to which soy milk should be used for the MCA assay. Thus, we decided to assess the effect of substrate concentration on the coagulant activity with different soy milks.

### 3.6. Effect of Substrate (Soy Milk Types) on Milk Clotting Activity

Several factors affect the milk clotting activity, among them, different types of substrates and enzyme concentrations which modify the rate of an enzyme-catalyzed reaction. When the enzyme concentration is small, the enzyme can completely combine with the substrate, and the degree of hydrolysis is increased. When more enzyme is added, its amount is higher than that of the substrate, resulting in a relatively small substrate concentration. Then, some amount of enzyme cannot combine with its substrate, leading to a modification in the enzymatic activity. 

**Figure 6:**
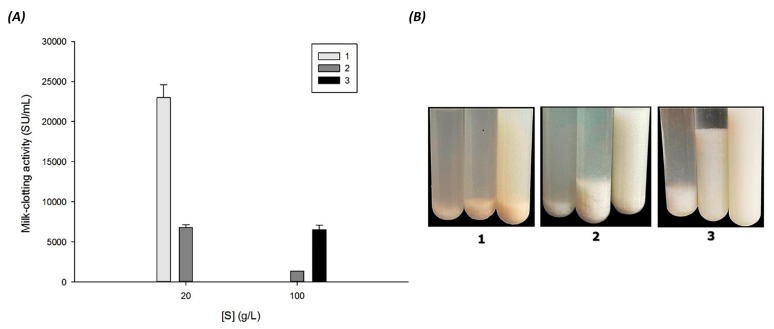
Milk-clotting activity from seed crude extract of *M. oleifera* on different types of commercial soy milk. (**A**): Left plot was drawn using the data obtained from the milk-clotting activity assay with different types of soy milk. (**B**): Right image shows the curd formation in test tubes. Substrate 1: AdeS; Substrate 2: Soyapac; Substrate 3: Soyalac. Asterisks represent statistical significance (three separate experiments) based on variance (one-way ANOVA), followed by HSD (*p* < 0.05 vs. Substrate 2: Soyapac).

Soy milk composition is variable, depending mainly of soybean varieties and processing methods [64]. Substrate specificity was determined using three different commercial soy milks (Figure 6). A high level of MCA was shown by substrate 1 (23,500 SU/mL) at 20 g/L and a moderated and poor MCA was revealed by substrates 2 and 3, respectively (Figure 6). Interestingly, seed extract activity is approximately 5 times higher using substrate 1 than using substrate 2. It is worth mentioning that when substrate 3 was used at low concentration (20 g/L), seed enzyme was not suitable to perform its activity. However, only at higher concentration (100 g/L of substrate 3), a good milk clotting activity of 6100 SU/mL was obtained, and it was even better than that obtained with skim milk at the same concentration (Figure 3). The differences between activities from all types of soy milk might be due to the difference in its physicochemical composition, mainly fats, proteins, and total solids in each kind of milk. Thus, seed extract possesses an exceptionally abundant and diverse specificity. Therefore, these results indicate that the milk clotting enzyme from *M. oleifera* seeds has a broad catalytic spectrum and suggests its usefulness for different applications in the dairy industry. Interestingly, our results indicated that Soyalac was the best substrate and hence it was selected for cheese production.

Numerous factors influence the primary and secondary coagulation phases as well as rheological properties to form gels. The most essential factors are protein (substrate) concentration, milk pH value, milk clotting enzyme type and concentration, calcium concentration, and temperature [59,67]. Earlier reports indicated that bovine milk clotting time is affected by type and protein content of the coagulant. Mehaia [68], for instance, indicated that clotting time of bovine milk can be longer when the concentration ratio of the protein content of the coagulant enzyme is increased because of the increased effectiveness of collisions due to a decreasing aqueous phase. Bruno et al. [69] also reported that a higher dilution of hieroymain fruit extract prolonged bovine milk clotting time. In contrast, a significant decrease in bovine milk clotting time was observed with an increase in the amount of *Solanum macrocarpon* extract [70]. Moreover, it was reported that MCA is increased when crude extract concentration of ginger rhizome is diluted [71].

### 3.7. Cheese Processing

Soy milk has been used as a cow milk alternative due to its high amounts of protein, iron, unsaturated fatty acids, and niacin but low amounts of fat, carbohydrates, and calcium compared with those of cow’s milk [72]. Various soy cheeses are made in some countries and have attracted increasing attention and have been used as a soft cheese-like product [73,74,75].

**Figure 7:**
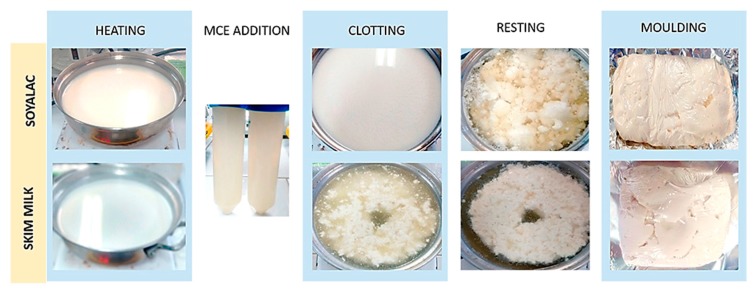
Workflow employed in cheese making using seed crude extract. The upper photos show soy milk cheese’s elaboration while the lower photos show skim milk cheese’s elaboration, both processed with Moringa seed extract. All experiments were performed in triplicate.

To further confirm the suitability of the *M. oleifera* seed extract as a rennet substitute in cheesemaking, the potential of the milk-clotting enzyme from seed extract was proved. A soft-white cheese was obtained with soymilk (Soyalac) while the cheese made with skim milk was hard and crumbly (Figure 7). The results demonstrated no significant differences in the coagulation time using both types of milk, and cheeses of 3.6 to 6.8 g were obtained.

Liu and Chang [76] reported that tofu texture made from soy milk is affected by several factors including soybean composition, soy milk processing, type and amount of coagulant, and processing methods. Glycinin and β-conglycinin are the major storage proteins (globulins) in soy foods. Glycinin corresponds to the 11S protein, and β-conglycinin is the principal component of the 7S protein. Tezuka et al. [77] reported that higher amounts of coagulant are required for the 7S globulin-rich soy milk than 11S globulin-rich or normal soy milks. The gel hardness depended mainly on glycinin content, hence, if the 11S/7S ratio increases, the hardness of gel is enhanced [78]. In accordance with this background, we assumed that commercial soy milks used in this study presented a low glycinin fraction and an enriched 7S-globulin content. However, additional studies are needed to know the quality and composition of the main protein present in commercial soy milks.

## 4. Conclusions

Leaf and flower enzymes displayed a negligible value of milk-clotting activity, whereas the seed enzyme demonstrated a high milk-clotting activity on whole, skim, and soy milks. In addition, it would be interesting to consider if other components, such as phytochemicals found in *M. oleifera* seed extract, could have antioxidant effects, which may help to reduce the risk of cardiovascular diseases. With these results it can be inferred that Mexican *M. oleifera* seed extract can be successfully used for different types of cheese manufacture with nutritional benefits, as well as several industrial applications.

## Figures and Tables

**Table 1:** Values of whole milk-clotting activity from the different parts of *M. oleifera*.

Crude Extract	Protein Concentration (mg/mL)	Total MCA (SU/mL)	Specific Activity (SU/mg Protein)
Seeds	10.30 ± 0.45	3419.26 ± 186.80	331.96
Flowers	10.62 ± 0.77	13.66 ± 1.12 **	0.77
Leaves	28.43 ± 3.04 *	ND	ND
Calf rennet	14.10 ± 0.95	6060.6 ± 0.71 *	429.82

The values are expressed as mean ± standard deviation. ND: None detected. MCA: milk-clotting activity. SU: Soxhlet unit. Asterisks represent statistical significance (three separate experiments) based on variance (one-way ANOVA), followed by Tukey’s honestly significant difference (HSD) (* *p* < 0.05 vs. Seeds and Flowers; ** *p* < 0.05 vs. Seeds).

**Table 2:** Nutritional composition of different soymilk (per 1 cup).

Commercial Milk Type	Fat (g)	Fiber (g)	Protein (g)	CHO * (g)	Ratio ^+^	Ca	Fe	P	Zn	Calories (Kcal)
[50]	4.67	3.18	6.73	4.43	0.69	9.8 mg	1.4 mg	120.05 mg	NR	79
AdeS	4.5	3.0	6.2	10.8	0.72	27%	9%	NR	22%	109
Soyalac	1.0	2.0	2.0	10.0	0.50	19%	21%	NR	NR	60
Soyapac	6.0	0	6.6	9.1	0.90	25%	13%	28%	25%	117

* Carbohydrates; ^+^ the fat to protein ratio; NR, not reported. Nutritional facts were taken from the package labels of each type of milk.
